# Structure of the SCAN Domain of Human Paternally Expressed Gene 3 Protein

**DOI:** 10.1371/journal.pone.0069538

**Published:** 2013-07-23

**Authors:** Vadim Rimsa, Thomas C. Eadsforth, William N. Hunter

**Affiliations:** Division of Biological Chemistry and Drug Discovery, College of Life Sciences, University of Dundee, Dundee, United Kingdom; Yale University School of Medicine, United States of America

## Abstract

Human paternally expressed gene 3 protein (PEG3) is a large multi-domain entity with diverse biological functions, including acting as a transcription factor. PEG3 contains twelve Cys_2_-His_2_ type zinc finger domains, extended regions of predicted disorder and at the N-terminus a SCAN domain. PEG3 has been identified as partner of the E3 ubiquitin-protein ligase Siah1, an association we sought to investigate. An efficient bacterial recombinant expression system of the human PEG3-SCAN domain was prepared and crystals appeared spontaneously when the protein was being concentrated after purification. The structure was determined at 1.95 Å resolution and reveals a polypeptide fold of five helices in an extended configuration. An extensive dimerization interface, using almost a quarter of the solvent accessible surface, and key salt bridge interactions explain the stability of the dimer. Comparison with other SCAN domains reveals a high degree of conservation involving residues that contribute to the dimer interface. The PEG3-SCAN domain appears to constitute an assembly block, enabling PEG3 homo- or heterodimerization to control gene expression in a combinatorial fashion.

## Introduction

Human paternally expressed gene 3 protein (PEG3) is a large multi-functional protein of nearly 1600 amino acids with a mass of about 165 kDa. The protein carries twelve zinc finger DNA-binding motifs, which are an important component of transcription [1]. These zinc fingers are classed as Cys_2_-His_2_ Krüppel-type motifs where metal ion coordination helps to stabilize a simple ββα fold [2]–[4]. The Krüppel-type fingers are separated by the Krüppel-link, a conserved sequence of seven amino acids [5]. An individual zinc finger can bind three-four base pairs in the major groove of DNA, but they often occur in tandem and work collectively to allow for recognition of longer DNA sequences [6], [7]. Consistent with the presence of a large number of zinc fingers, PEG3 has been shown to bind DNA in a sequence specific manner [8], and has been detected predominantly in the nucleus, supporting the notion it is a *bone fide* transcription factor [1], [9]. In addition, PEG3 is reported to be present in the cytoplasm where it interacts with proteins such as tumour necrosis factor receptor-associated factor 2 (TRAF2) to regulate the NF-κB signal transduction pathway, and with the E3 ubiquitin-protein ligase seven-in-absentia homolog one (Siah1a) to promote p53-mediated apoptosis [10], [11]. PEG3 also binds β-catenin to induce its degradation, thereby inhibiting the Wnt signalling pathway [12]. Whole animal studies have revealed major physiological changes in mice following targeted mutation of the *peg3* gene. When a knockout model was assessed, growth retardation, increased levels of body fat, impairment of maternal behaviour and changes in male sexual behaviour were noted [13]–[16]. Such pleiotropic effects identify its importance, however the precise molecular mechanism whereby PEG3 contributes to distinct aspects of biology remains poorly understood.

Proteins that carry Cys_2_-His_2_ Krüppel-type motifs frequently contain other domains, which are implicated in subcellular localization, protein-protein and protein-DNA interactions. These include the Krüppel-associated box domain, the poxvirus and zinc finger (POZ), alternatively known as the BTB (broad-complex, tram track, and bric-a-brac), and the SCAN domain, sometimes called the leucine rich motif [17]–[21]. The name, SCAN, is derived from the first letters of four founding members of the family (SRE-ZBP, Ctfin51, AW-1 (ZNF174), and Number 18) [20]. PEG3 contains a SCAN domain near the N-terminus. The domain is a small, typically 80 to 90 residues, highly conserved structure. The human genome encodes 71 SCAN domains, 64 of which appear within functional genes [22]. The domain functions as a protein-protein interaction motif, mediating self-interaction and binding other proteins [23]–[25]. The ability of a SCAN domain to self-associate was first shown for ZNF174 using a mammalian two-hybrid system and subsequently heterodimer formation was also noted [23]. Our current understanding suggests that the presence of SCAN domains in some proteins carrying Cys_2_-His_2_ Krüppel-type motifs allows them to interact in a combinatorial fashion to control gene expression [26].

Previously, two NMR and two crystal structures of SCAN domains have revealed the structural topology, which is constructed around five *α-helices* [26]–[28]. The SCAN domain forms a dimer with structural homology, appearing like a domain-swapped assembly, to the C-terminal domain (CTD) of the retroviral capsid protein [27], which is critical for capsid dimerization and viral assembly [29].

Here, we report the structure of the isolated SCAN homodimer from the human PEG3 protein (PEG3-SCAN). Comparisons with other SCAN structures in the Protein Data Bank (PDB) and the importance and contributions of residues involved in PEG3-SCAN dimerization are discussed, and provide insight into homo- and heterodimer assembly.

## Materials and Methods

### Protein Expression and Purification

The gene encoding the SCAN domain of human PEG3 (amino acids 40–130; UniProt entry Q9GZU2) was purchased in the pUC57 vector (GenScript). The gene was subsequently sub-cloned into a modified pET15b vector (Novagen), designed to express the protein of interest with an N-terminal hexa-His tag and a Tobacco Etch Virus (TEV) protease cleavage site. The sequence-verified clone (DNA Sequencing Unit, University of Dundee) was transformed into *Escherichia coli* Bl21 (DE3) Gold cells (Novagen) for expression. Cells were grown in Luria-Bertani media at 37°C to an OD_600_ of ∼0.6, followed by induction with 0.2 mM IPTG. Cells were then grown overnight at 22°C and harvested by centrifugation at 3,500 g for 30 minutes at 4°C. The cell pellet was suspended in lysis buffer (50 mM Tris-HCl, pH 7.5, 150 mM NaCl, 20 mM imidazole) containing DNase I (0.1 mg), with a single protease inhibitor tablet (Roche) and lysed using the French Press (16,000 psi). The extract was centrifuged at 37,500 g for 30 minutes at 4°C to pellet insoluble debris. The supernatant was loaded onto a 5 mL HisTrap HP column (GE Healthcare) and a linear imidazole concentration gradient from 20 mM to 1 M was applied. The PEG3-SCAN sample eluted from the column at approximately 200 mM imidazole. Fractions containing the recombinant protein were pooled and dialyzed in buffer (50 mM Tris-HCl, pH 7.5, 150 mM NaCl), prior to addition of TEV protease to remove the His-tag. TEV protease was prepared in-house by Keri Barrack (University of Dundee). 1 mg of TEV was used per 20 mg of protein in a cleavage reaction, which was performed at room temperature for three hours. After TEV digestion, three non-native residues (Gly-His-Met) remained at the N-terminus of the product. The protein was passed through a second nickel-affinity column, which removed uncleaved protein and the His-tagged TEV protease. Gel filtration (GF, Superdex 75 16/60 column; GE Healthcare) was used as a final purification step. The column had previously been calibrated with molecular weight standards, blue dextran (>2,000 kDa), thyroglobulin (669 kDa), ferritin (440 kDa), aldolase (158 kDa), conalbumin (75 kDa), ovalbumin (43 kDa), carbonic anhydrase (29.5 kDa), ribonuclease A (13.7 kDa) and aprotinin (6.5 kDa). PEG3-SCAN eluted from the size exclusion column as a single symmetric peak with a mass of approximately 28 kDa. The theoretical mass of PEG3-SCAN is 11,434 Da; therefore PEG3-SCAN forms a homodimer in solution. The purity of the sample was checked further by SDS-PAGE and mass spectrometry (Fingerprint Proteomics Facility, University of Dundee). The single protonated species as observed by mass spectrometry was 11,432 Da, in close agreement with the theoretical mass. Protein concentration was determined spectrophotometrically using a theoretical molar extinction coefficient of 16,960 M^−1^ cm^−1^ [30].

The gene coding for part of human Siah1 without the RING domain (amino acids 91–282; UniProt entry Q8IUQ4) was purchased in the pUC57 vector (GenScript). The gene fragment was transferred into the pET15b vector (Novagen) for recombinant expression. Siah1 and ^15^N-labeled Siah1 for NMR studies were prepared and purified using a similar protocol to that for PEG3-SCAN, except that isotopically enriched Siah1 was expressed in the minimal media. A single sharp peak was observed for Siah1 on GF column with a mass of around 39 kDa. This value matches closely to the weight of Siah1 homodimer, as the theoretical mass of a monomer is 21,897 Da. The presence of the Siah1 homodimer was confirmed by size exclusion chromatography (SEC) coupled with multi-angle light scattering. This supports previous studies showing Siah1 is a dimeric protein [31]. The purity of the sample was also analyzed by SDS-PAGE and mass spectrometry, which showed a single protonated species of 21,875, closely matching the theoretical size. Protein concentration was determined by UV spectrophotometry using a theoretical extinction coefficient of 22,960 M^−1^ cm^−1^ [30]. The association between PEG3-SCAN and Siah1 was tested in SEC by combining protein samples together at one-to-one stoichiometry in 50 mM Tris-HCl, pH 7.5, and 150 mM NaCl buffer. The mixture was left overnight at 4^o^C, before it was run on a GF column (Superdex 75 16/60 column; GE Healthcare). The NMR experiment was done under the following conditions, where 100 µM of ^15^N-labeled Siah1 was mixed with 100 µM of unlabeled PEG3 in 50 mM HEPES, pH 7.5, 50 mM NaCl, and 5% D_2_O buffer. The chemical shift perturbations in the ^1^H-^15^N HSQC of Siah1 were monitored upon addition of PEG3.

### Thermal Stability, Crystallization and Data Collection

Differential scanning fluorimetry (DSF) was used to investigate the influence of different buffers on the thermal stability of the samples. DSF suggested the presence of a globular SCAN domain, which displayed a melting temperature of 52°C in a number of buffers. Since no buffer appeared to enhance stability the protein was left in the GF buffer (50 mM Tris-HCl, pH 7.5, 150 mM NaCl). The melting temperature of Siah1 was 64^o^C in the buffers tested again with a profile indicative of a folded protein.

**Figure 1:**
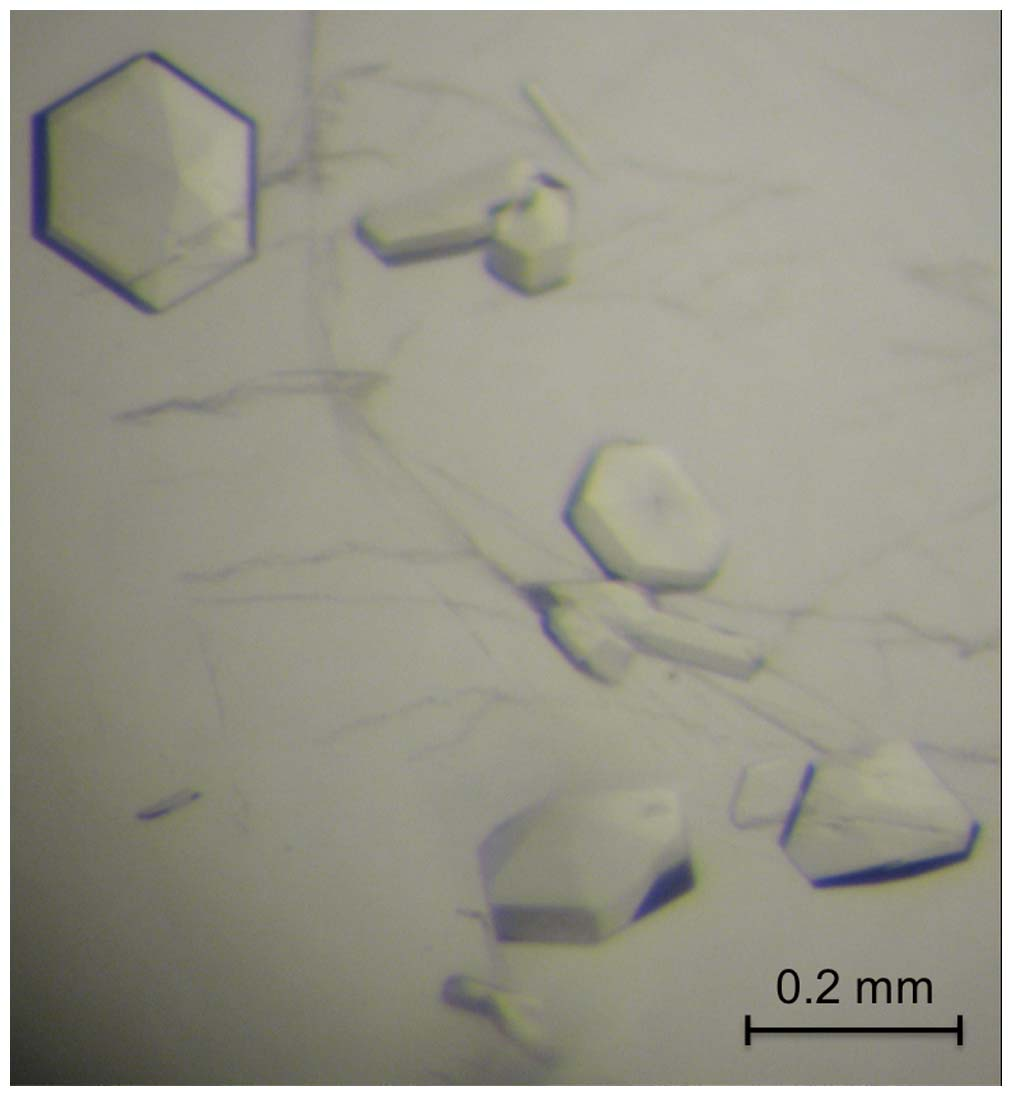
Crystals of PEG3-SCAN. Crystals grown in 50 mM Tris-HCl pH 7.5 and 150 mM NaCl.

Crystallization screening of PEG3-SCAN was carried out with the high-throughput Phoenix liquid handling system (Art Robins Instruments/Rigaku) and several commercially available screens (Hampton Research). 100 nL of protein sample at ∼16.5 mg mL^−1^ were mixed in sitting-well plates with 100 nL of reservoir solution against 70 µL of the same reservoir solution. Crystals appeared within a day in numerous conditions that contained polyethylene glycol (PEG) of different molecular weight. These crystallization conditions were scaled up from these nano-drops to micro-drops of total volume 4 µL. However, the rod-shaped crystals that were observed were small, approximately 0.1×0.02×0.02 mm, and gave poor diffraction. While preparing more protein for use in crystal optimization it was observed that larger crystals actually formed spontaneously when the protein was concentrated in the GF buffer using Vivaspin 20 concentrators with a 3,000 MW cut off (Sartorius Stedim Biotech). Hexagonal bipyramid crystals, reaching 0.2×0.2×0.2 mm dimensions, formed within minutes (Fig. 1). The average protein concentration in the centrifugal device was 5 mg mL^−1^, but likely to have been considerably higher near the membrane where crystal nucleation occurred. The selection of a suitable cryoprotectant required extensive screening and optimization. The use of glycerol, ethylene glycol and paratone-N produced either poor diffraction or pronounced ice rings. The most favourable cryoprotectant was PEG200. Crystals were transferred into cryo-solution of PEG200 and GF buffer at 1∶1 ratio prior to flash cooling for X-ray diffraction studies.

### Structure Solution and Refinement

**Figure 2:**
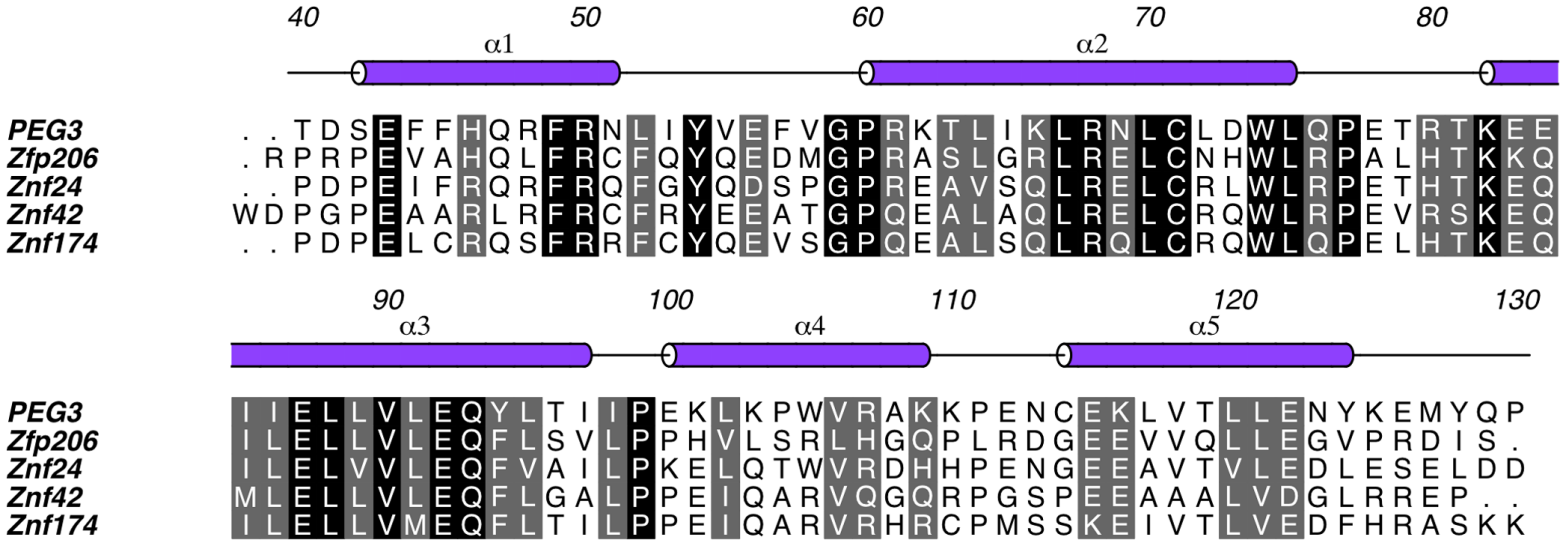
The primary and secondary structure of PEG3-SCAN. Five α-helices are shown as cylinders (purple) and are numbered accordingly. Multiple sequence alignment of PEG3-SCAN with other SCAN proteins from PDB was performed with ClustalW2 [48]. PEG3-SCAN residues that are strictly conserved in Zfp206 (PDB: 4E6S), Znf24 (PDB: 3LHR), Znf42 (PDB: 2FI2) and Znf174 (PDB: 1Y7Q) are encased in black, while residues sharing similar properties in five proteins are encased in grey. The numbers that are shown above the secondary structure mark residues in the full length PEG3 protein (UniProt: Q9GZU2).

Diffraction data were collected in-house with a Micromax-007 rotating anode generator using CuKα (λ = 1.5414 Å) radiation and an AFC11 Saturn 944+ CCD detector (Rigaku). The data were indexed and integrated with *iMOSFLM* [32] and scaled with *AIMLESS* from the *CCP4* program suite [33]. The structure was solved by molecular replacement with *PHASER* [34] using a poly-Ala model of the SCAN domain dimer from the mouse zinc finger protein 206 (Zfp206, PDB code 4E6S [26]) that shares 38% sequence identity with the PEG3-SCAN domain. The output model was subjected to a round of rigid body and restrained refinement using *REFMAC5* [35]. The poly-Ala model was modified to the sequence of human PEG3-SCAN based on inspection of electron and difference density maps in *COOT* [36]. Several residues and side chains for which there was no convincing electron density were deleted. Additional rounds of restrained least-squares refinement followed, interspersed with map inspection and model manipulation. The refinement used the automatic geometry and *B*-factor restraint weights. Neither non-crystallographic symmetry (NCS) restraints nor TLS (Translation/Libration/Screw) were used in refinement. A number of ligands (ethylene glycol, diethylene glycol and triethylene glycol) were included in the model on the basis of the difference density and chemical environment, and refined successfully. These molecules are likely to be decomposition products or impurities of the PEG200 cryoprotectant. The final model also includes multiple side chain conformers and water molecules. The stereochemistry of the model was checked using *MOLPROBITY* [37]. *PISA* (Protein Interfaces, Surfaces and Assemblies [38]) was used to calculate surface and dimer interface areas. Fig. 2 was prepared with *ALINE* [39] and the others were produced using *PyMOL* [40]. Data collection and structure refinement statistics are shown in Table 1. The atomic coordinates and structure factors have been deposited in the PDB with accession code 4BHX.

**Table 1:** Crystallographic statistics.

	PEG3-SCAN
Space group	*P*6_5_
Unit cell dimensions: *a*, *b*, *c* (Å)	83.61, 83.61, 55.23
Resolution^a^ (Å)	13.8–1.95 (2.00–1.95)
No. of reflections	453776 (23734)
Unique reflections	16090 (1143)
Completeness (%)	99.7 (99.8)
Multiplicity	28.2 (20.8)
<*I*/σ*I*>	38.2 (9.7)
Wilson *B* (Å^2^)	20.6
Mosaicity (°)	0.5
**Residues**	
Chain A	40–127
Chain B	40–129
Water/ethylene glycol/diethylene glycol/triethylene glycol	155/21/4/2
*R_merge_* b (%)	7.0 (31.8)
*R_work_* c (%)	17.15 (19.0)
*R_free_* d (%)	22.38 (24.4)
***Mean B*** **-factors (Å^2^)**	
Chain A	27.4
Chain B	25.9
Waters	38.4
Other ligands	43.2
R.m.s.d. bond lengths (Å)	0.02
R.m.s.d. bond angles (°)	2.02
**Ramachandran plot** (%)	
Most favoured	98.4
Additional allowed	1.6
Outliers	0.0

a Values in parentheses refer to the highest resolution shell (2.00–1.95 Å).

b *R_merge_* = ∑*h∑i||*(*h,i*)–<I(*h*)> ∑*h∑i* I(*h,i*); where I(*h,i*) is the intensity of the *i*th measurement of reflection *h* and <I(*h*)> is the mean value of I(*h,i*) for all *i* measurements.

c *R_work_* = ∑*hkl*||*F_o_*|−|*F_c_*||/∑*|F_o_|,* where *F_o_* is the observed structure factor amplitude and the *F_c_* is the structure-factor amplitude calculated from the model.

d *R_free_* is the same as *R_work_* except calculated with a subset, 5%, of data that are excluded from refinement calculations.

## Results and Discussion

### Structure Quality

Crystals of human PEG3-SCAN belong to space group *P*6_5_, with a V_M_ value of 2.44 Å^3^ Da^−1^ and solvent content of approximately 50% for an asymmetric unit comprising two polypeptide chains. The polypeptides are arranged as a symmetrical dimer consistent with the GF results obtained during protein purification and also with previously solved structures of SCAN domains. The crystals diffract to a resolution of 1.95 Å and the majority of the residues are located within well-defined electron density, apart from a few residues at the C-terminus. In addition, the final model contains two extra residues (His and Met) at the N-terminus, which are remnants from proteolytic cleavage of the histidine tag. A Ramachandran plot indicates that 98.4% of the amino acids are located in the most favoured region with no outliers.

### Overall Structure

**Figure 3:**
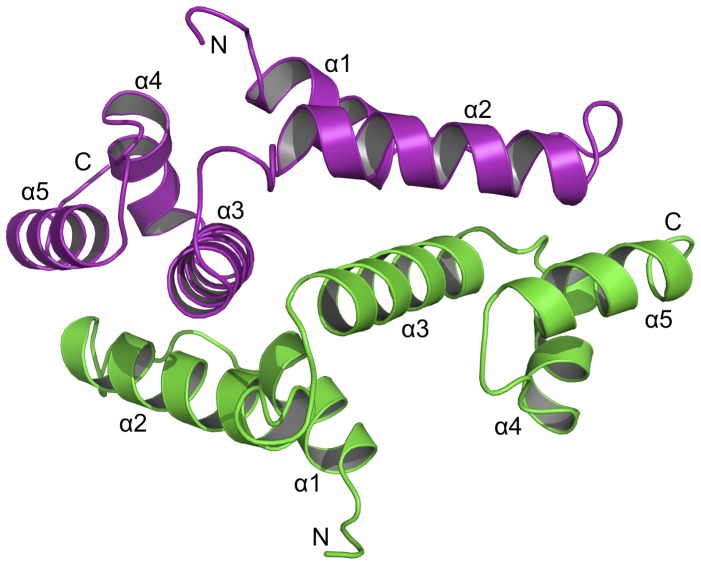
Overall structure of PEG3-SCAN. The homodimer is shown as ribbons with one subunit green, the partner purple. The N- and C- termini as well as the five α-helices of each monomer are labeled accordingly.

The human PEG3-SCAN domain folds as an extended V-shaped structure, with approximate overall dimensions of 50×25×25 Å. Each arm of the V-shape is approximately 35 Å in length. The subunit comprises five α helices and the assignment of secondary structure onto the sequence is presented in Fig. 2 with the fold depicted in Fig. 3. Helices α1 and α2 which form an N-terminal sub-domain are aligned antiparallel to create one half of the V. A C-terminal sub-domain, which forms the other half, results from α3, α4 and α5 being packed together. The domain aligns with the N-terminal sub-domains interacting with partner C-terminal sub-domains (Fig. 3) to form a dimer with approximate dimensions of 50×37×30 Å. A least-squares superposition of subunits with *LSQKAB* [41] gives an r.m.s.d. (root-mean-square deviation) of 0.57 Å for 90 Cα atoms, which shows there are no major conformational differences between the two subunits. It is noteworthy that such a low value was obtained in the absence of NCS restraints.

The total surface area of a subunit, calculated with *PISA* [38], is approximately 7400 Å^2^ of which 1700 Å^2^ are buried within the dimer. Therefore, about 23% of the surface area of each monomer is involved in dimerization. The free energy of dissociation (ΔG^diss^) is estimated as 19.4 kcal mol^−1^, and suggests that this assembly is thermodynamically stable, consistent with the observation of a stable dimer in solution. Similar values are observed for other SCAN structures. For example, the interface area and ΔG^diss^ for the Znf24 dimer (PDB code 3LHR) are 23% and 21.8 kcal mol^−1^, respectively.

**Figure 4:**
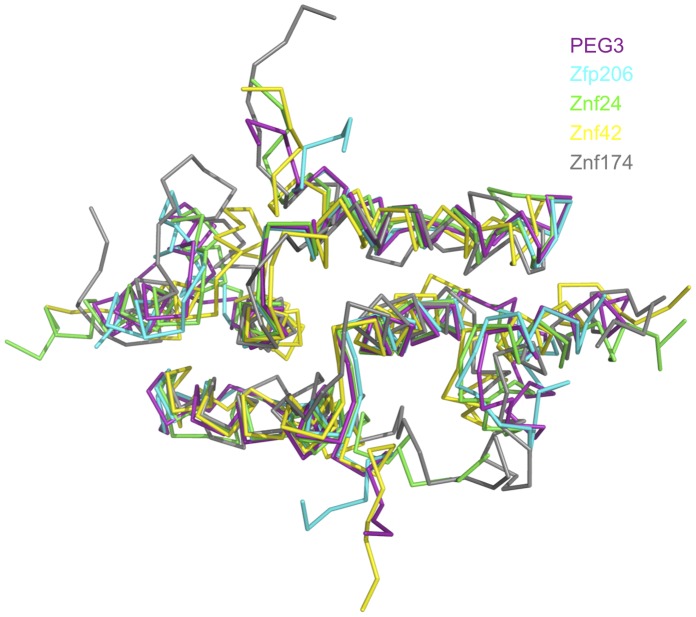
Superposition of the PEG3-SCAN homodimer (purple) with other SCAN structures. Zfp206 (PDB 4E6S), Znf24 (PDB 3LHR), Znf42 (PDB 2FI2) and Znf174 (PDB 1Y7Q) are shown in cyan, green, yellow and grey, respectively. Superposition was calculated using secondary structure matching [49].

At present there are four SCAN domain structures in the PDB, two crystal structures and two determined by solution NMR. Sequence conservation of these four with human PEG3-SCAN is presented in Fig. 2. The superposition of the PEG3-SCAN dimer onto these other dimers reveals an overall structural conservation (Fig. 4), with calculated r.m.s.d. values presented in Table 2. The largest deviations among SCAN structures occur at the N- and C-terminal ends, which show higher flexibility than the core, and α4, which is positioned away from the dimer interface. The r.m.s.d. values for alignments with the SCAN domain dimers of Znf42 and Znf174 show higher variation, more than 1.0 Å greater, than for the X-ray structures, because of the greater uncertainties associated with the NMR structures and that the fit involves an average of 20 conformers that represent their NMR derived structures.

**Table 2:** Structure and sequence similarity between PEG3-SCAN and other SCAN domains.

Protein name	PDB codes	R.m.s.d (Å)	R.m.s.d alignment length	Sequence identity (%)
Zfp206	4E6S	1.57	157	38
Znf24	3LHR	1.51	164	48
Znf42	2FI2	2.85	155	35
Znf174	1Y7Q	2.87	167	43

These included crystal structures of Zfp206 and Znf24, and solution NMR structures of Znf42 and Znf174. R.m.s.d. calculations were carried out with PDBeFold using secondary structure matching [49] with the PEG3-SCAN dimer in the superposition. Sequence alignment was performed with ClustalW2 using residues 40–130 of the full-length PEG3 against the core of the SCAN domain, as well as 2–5 flanking residues, of other proteins.

### Residues Forming the SCAN Dimer Interface

**Figure 5:**
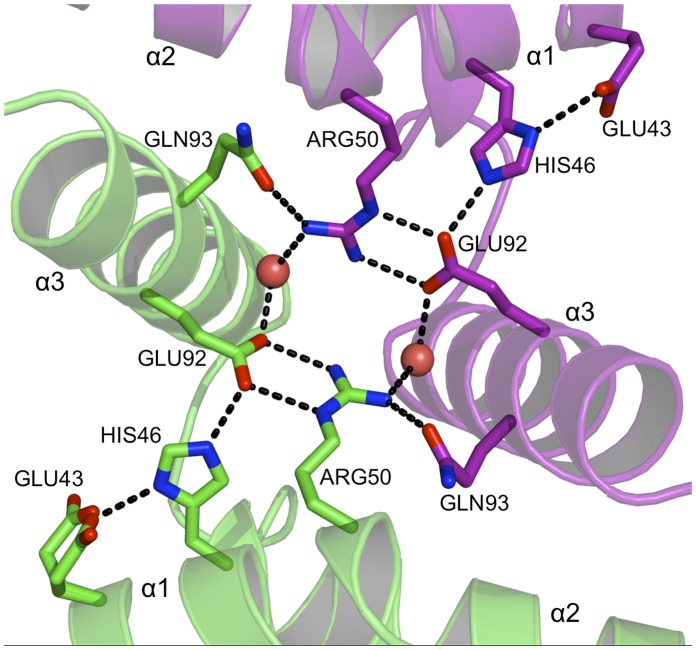
The dimer interface of PEG3-SCAN (I). A hydrogen-bonding network is formed between conserved residues lining the subunit-subunit interface. Water molecules are shown as red spheres, N and O positions are colored blue and red respectively, C positions are purple or green depending on the subunit to which they belong, hydrogen bonds are depicted as dashed lines. The same color scheme is used in Figures 6, 7 and 8.

**Figure 6:**
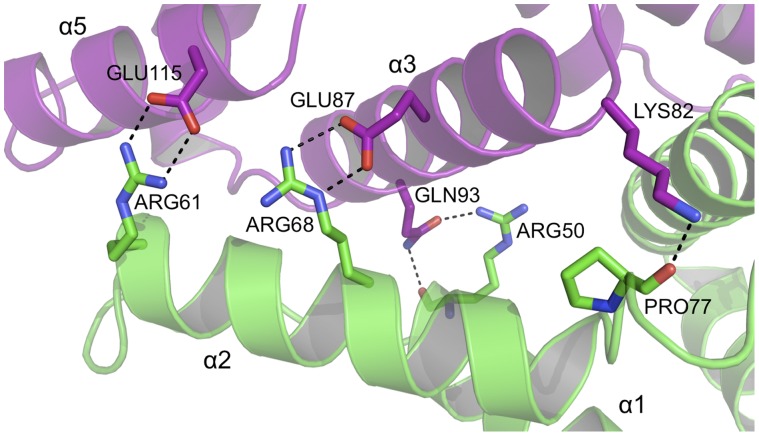
The dimer interface of PEG3-SCAN (II). A second cluster of hydrogen bonding and salt bridge interactions at the subunit-subunit interface.

The human PEG3-SCAN homodimer is held together by an extensive network of hydrogen-bonding, salt-bridge interactions and van der Waals forces. Even though the overall sequence identity among the five known SCAN structures is only 40–50% (Fig. 2), the key residues located at the dimer interface and that contribute to inter-subunit associations are conserved. The majority of these intermolecular contacts are formed between α1 and α2 (the N-terminal sub-domain) of one subunit and α3 on the C-terminal sub-domain of the partner. Helices α2 and α3 show the highest amino acid conservation when comparing the sequences of these known SCAN domain structures and the conserved residues contribute significantly to either direct inter-subunit associations or organising the side chain positions to facilitate such interactions. For example, a hydrogen bond linking Glu43 with His46 helps to position the imidazole to also interact with Glu92 (Fig. 5). Glu92, close to the position of the NCS two-fold axis, forms a salt bridge interaction with Arg50 and also a well ordered and buried water molecule. Arg50 then donates a hydrogen bond to the partner subunit Gln93 and the water molecule mediates contact between Arg50 and the partner subunit Glu92. Arg68 forms an inter-subunit salt bridge with Glu87, so linking α2 of one subunit with α3 of the partner subunit (Fig. 6). These residues are highly conserved within SCAN domains and observed to form similar hydrogen bonding patterns where the structures are known. Furthermore, mutation of both the equivalent Arg50 and Arg61 residues to alanines in Zfp206 destabilizes heterodimerization with Zfp110 [26]. Thus, these invariant residues seem to play an important role in both homo- and heterodimerization. Other residues establishing inter-subunit contacts include Lys82, which interacts with Pro77 and Arg61 with Glu115 (Fig. 6). The former makes a hydrogen bond, while the later makes a salt-bridge interaction, which links α2 with the partner subunit α5. The later pair of residues is substituted to a similar lysine glutamine pair in some SCAN domain sequences.

**Figure 7:**
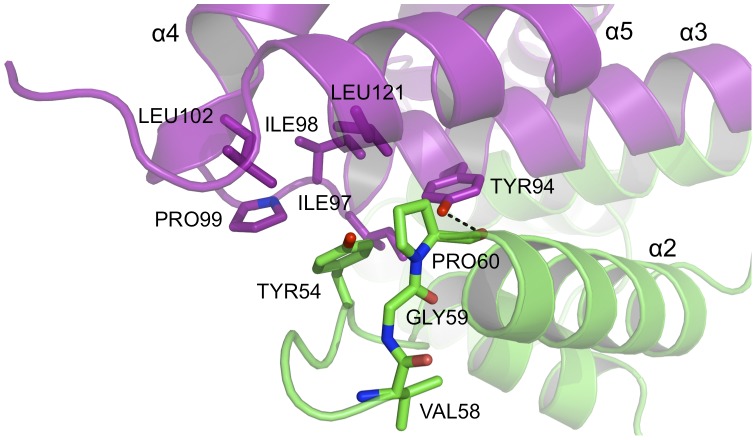
The dimer interface of PEG3-SCAN (III). A pronounced hydrophobic patch occurs at each end of the assembly to stabilize the dimer. The conserved Tyr94 extends across the dimer interface, contributes to hydrophobic interations and donates a hydrogen bond to the carbonyl of Pro60.

Some of the interactions noted in the PEG3-SCAN dimer are absent from the other structures. For example, a hydrogen bond donated from the side chain of Tyr94 on one subunit to the carbonyl of Pro60 (Fig. 7) on the partner cannot occur in other structures where phenylalanine replaces the tyrosine. Furthermore, in PEG3-SCAN there is an inter-subunit salt bridge between Glu56 and Lys101 (data not shown). Glutamate replaces the lysine in most other SCAN domains. Such sequence variations may confer a preference of different SCAN domains to form distinctive homo- and heterodimers.

Whilst most of the dimer interface excludes water molecules, with the notable exception described above, at the periphery of the SCAN dimer there are five that are involved in mediating subunit-subunit interactions (data not shown). This does not appear to be a major factor in stabilizing the dimer given the high percentage of the surface area involved in direct association as described above.

**Figure 8:**
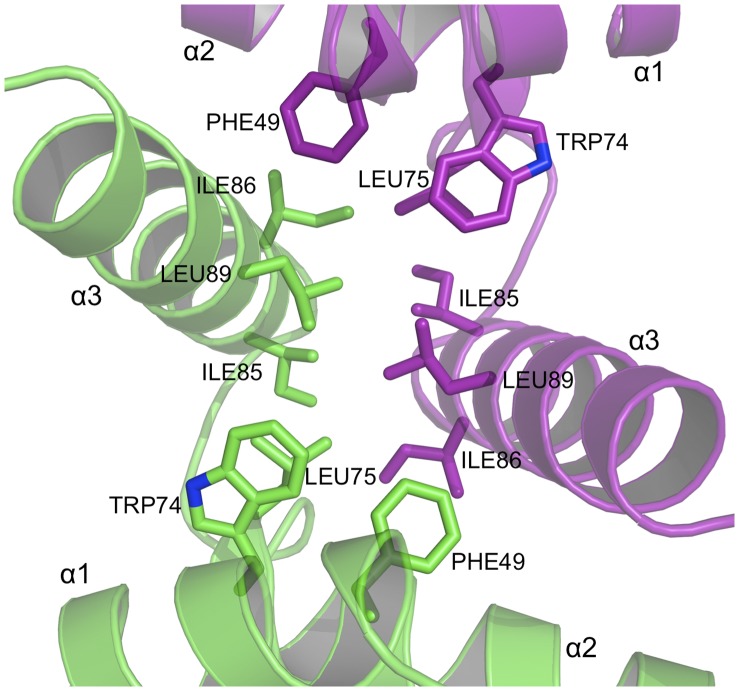
The dimer interface of PEG3-SCAN (IV). A group of conserved, aliphatic and aromatic residues form a hydrophobic core to stabilize the dimer.

The dimer interface includes important stabilizing contributions from hydrophobic residues. A hydrophobic patch comprising Phe49, Trp74, Leu75, Ile85, Ile86 and Leu89, from α2 and α3, make van der Waals interactions to equivalent residues in the partner subunit (Fig. 8). In another area, a conserved Tyr54 and Pro60 from one subunit interact with a hydrophobic core formed by Tyr54, Ile97, Ile98, Pro99, Leu102 and Leu121 from α3 and α5 of the partner subunit (Fig. 7). Other hydrophobic residues extending across the dimer interface include Leu52, Leu64, Leu67, Val90 and Val118 (not shown).

The strictly conserved pair of residues Pro60 and Pro99, already mentioned as contributing to the inter-subunit hydrophobic associations, are positioned at the start of α2 and α4 respectively (Fig. 7), and likely important to initiate formation of the two helices.

### Function of the SCAN Domain

Transcription factors form complexes that regulate the expression of target genes. For example, the proto-oncogene Jun, belonging to the basic region-leucine zipper (bZIP) family, regulates gene transcription in a specific manner by forming homo- and heterodimers. It is the combination of dimerization state and binding partners that determine activity and DNA-binding site preferences [42]. Similar control of transcription has been observed in some Cys_2_-His_2_ zinc finger containing transcription factors that contain a protein-protein binding BTB/POZ domain. Self-oligomerization of this domain in the GAGA transcription factor promotes DNA binding affinity and specificity [43]. The SCAN domain may be playing a similar regulatory role in mammalian gene expression by mediating both homo- and selective hetero-dimerization of Cys_2_-His_2_ zinc finger proteins. To date, there are no data to suggest that SCAN domains participate directly in transcriptional activation or repression. The possibility that PEG3 could homodimerize through the SCAN domain suggests however that PEG3 binds to at least some target genes as a homodimer. In particular, this might be a prerequisite for PEG3 binding palindromic DNA motifs. Consistent with this is the finding that the Zfp206 transcription factor can form a homodimer using its SCAN domain and binds to a palindromic sequence [26], [44]. A 15 base pair non-palindromic DNA binding motif for PEG3 was predicted and subsequently validated using electromobility shift and promoter assays [8]. Although the full-length motif was required for maximal binding, PEG3 was observed to bind to the partial sequence as well as to other regions with degenerate sequence.

The DNA binding selectivity and affinity of PEG3 might depend on its specific isoform. To date, there are four known PEG3 isoforms. One isoform contains only two Cys_2_-His_2_ zinc finger motifs, while in two other isoforms the SCAN domain is absent. Thus, the presence or absence of the SCAN domain and varying number of zinc finger motifs provides a potential additional control mechanism. A number of SCAN domain members have been reported to selectively interact with other SCAN members [23]–[26]. However, it has not been shown that PEG3-SCAN can form heterodimers. Superposition of a PEG3-SCAN subunit on the Zfp206 dimer structure shows a close fit and spatial conservation of key residues forming the intermolecular contacts (Fig. 2, Fig. 4, data not shown), suggesting the possibility that the PEG3-SCAN domain might form a heterodimer with Zfp206 as well as with other SCAN domain containing proteins. Future studies, focusing on the binding partners of PEG3-SCAN are required to inform on the role of PEG3 in gene regulation.

It has been suggested that some SCAN domains act as specific cellular localization sequences. For example, the SCAN domain of Zfp42 is reported to be essential for targeting the protein to promyelocytic leukemia nuclear bodies in the cell nucleus [45]. However, since localization studies using different constructs of PEG3 have indicted that the SCAN domain was not required for nuclear localization [12] then such a general function is unlikely.

It is not known whether SCAN domains interact with any other protein motifs in addition to self-association with other SCAN members. The function of PEG3 in regulation of TNF and Wnt signal transduction pathways has been implied by an ability to bind TRAF2, the E3 ubiquitin ligase Siah1 and β-catenin [10]–[12]. A yeast two-hybrid screen showed residues outside the SCAN domain (residues 268–502) to be required for interaction with full-length TRAF2 as well as a Siah2 fragment missing the RING (really interesting gene) domain (residues 106–326) [10]. The same study reported the interaction of a mouse PEG3 with Siah proteins by immunoprecipitation, while the later experiments using deletion generated constructs of human PEG3 revealed that the N-terminus including the SCAN domain (residues 1–268) were required for binding the full-length Siah1. Many proteins that interact with Siah1 contain the consensus Siah1 binding motif Val-x-Pro [46], [47]. This amino acid sequence is also present within the SCAN domain of PEG3, Val58-Gly59-Pro60, on a short segment of extended structure leading to α2 (Fig. 7). We sought to investigate whether the human PEG3-SCAN domain (residues 40–130) interacted with human Siah1. A construct of Siah1 without the RING domain (residues 90–282) was cloned, expressed and purified separately and combined with PEG3-SCAN in one-to-one stoichiometry. The sample was analyzed on a size exclusion column, but there was no evidence of complex formation (data not shown). An NMR study revealed no differences in chemical shifts in the two-dimensional ^1^H–^15^N HSQC (heteronuclear single quantum coherence spectroscopy) spectra of ^15^N-labeled Siah1 upon addition of unlabeled PEG3-SCAN (data not shown) indicative of a lack of interactions between the polypeptides. The lack of an association suggests that residues outside the SCAN domain might be required for interaction with Siah1 but there is no other obvious Siah1 binding motif. The PEG3-SCAN structure reveals that the Val58-Gly59-Pro60 motif is an extended conformation immediately prior to α2. The proline is buried and involved in inter-subunit interactions, and the valine side chain tucked down towards the side of α2, away from the surface of the protein. We speculate that if this is indeed a recognition site for Siah1 then conformational changes might be required to allow for complex formation. The conditions under which we investigated the potential interaction may not have been correct to allow such changes or alternatively the presence of the Val-x-Pro sequence is coincidence. Either way, our observations suggest that the data invoking Siah1 interactions with PEG3 should be re-evaluated.

### Concluding Remarks

In summary, we have determined the structure of SCAN domain from PEG3, a predicted transcription factor, and compared it with available SCAN domain structures. Our results show this domain forms a stable homodimer and we provide an analysis of the residues forming the dimer interface. The sequence alignment and an overlay of PEG3-SCAN with available SCAN domain structures shows overall structural conservation and serves to identify key residues important to the creation of the PEG3-SCAN dimer interface.

The SCAN domain of PEG3 appears to function as a convenient dimerization domain. Gel filtration chromatography and NMR studies revealed no interaction between the SCAN domain and the potential PEG3-binding protein Siah1. Future studies will be needed to determine if indeed the SCAN domain of PEG3 interacts with other SCAN family members as well as other protein motifs. The validation of binding partners would represent a crucial step towards unraveling the biological role of PEG3 itself.
